# COVID-19 Response in Sub-Saharan Africa: Lessons From Uganda

**DOI:** 10.1017/dmp.2020.248

**Published:** 2020-07-15

**Authors:** Ivan Lumu

**Affiliations:** Infectious Diseases Institute, Makerere University, Kampala, Uganda; and School of Medicine and Veterinary Medicine, University of Edinburgh, Edinburgh, UK

**Keywords:** COVID-19, Ebola, response, SARS CoV-2

## Abstract

The prolongation of the Ebola epidemic may have allowed some countries to prepare and respond to the coronavirus disease (COVID-19) outbreak. In Uganda, the surveillance structure built for Ebola virus disease (EVD) has become a pillar in the COVID-19 response. This testing and tracing apparatus has limited disease spread to clusters with zero mortality compared with the neighboring East African countries. As more sub-Saharan countries implement social distancing to contain the outbreak, the interventions should be phased and balanced with health risk and socioeconomic situation. However, having a decision-making matrix would better guide the response team. These initial lessons from EVD-experienced Uganda may be helpful to other countries in the region.

The coronavirus disease (COVID-19) pandemic, which started at a time when the second most prolonged Ebola virus disease (EVD) outbreak was nearly declared to have ended, has left many wondering whether sub-Saharan Africa was ready for the pandemic. The prolongation of the Ebola outbreak has allowed some countries like Uganda and Congo the opportunity to enhance their abilities to prepare and respond to COVID-19.^1^ When the outbreak was declared, Uganda swiftly moved to reorient its testing and contact tracing apparatus from EVD surveillance to COVID-19. Screening measures at the airport previously developed for EVD helped detect the first case on March 21, 2020. By March 24, 2020, 203 samples from travelers had been tested, 14 of which were positive for the virus, severe acute respiratory syndrome coronavirus 2 (SARS-CoV-2) (Figure 1A). Interestingly, only 5 of the 14 confirmed cases were detected by symptom screening at the airport. The others were low-risk travelers who had been allowed home, an indication that some asymptomatic cases were already in the community. Due to the inefficiency of thermal screening in detecting asymptomatic patients,^2^ it was abandoned. Instead, the travel manifest of March was retrieved to guide testing, contact tracing, isolation, and quarantine. By March 31, a total of 1370 samples had been tested, from which 44 were confirmed and isolated, and 660 contacts along with 1015 travelers were identified and quarantined. To date, testing and tracing numbers remain high in Uganda compared with the country’s politically stable East African neighboring countries^3^ without EVD experience (Table 1). This testing and tracing apparatus has limited disease spread to clusters with zero mortality.^3^

**FIGURE 1:**
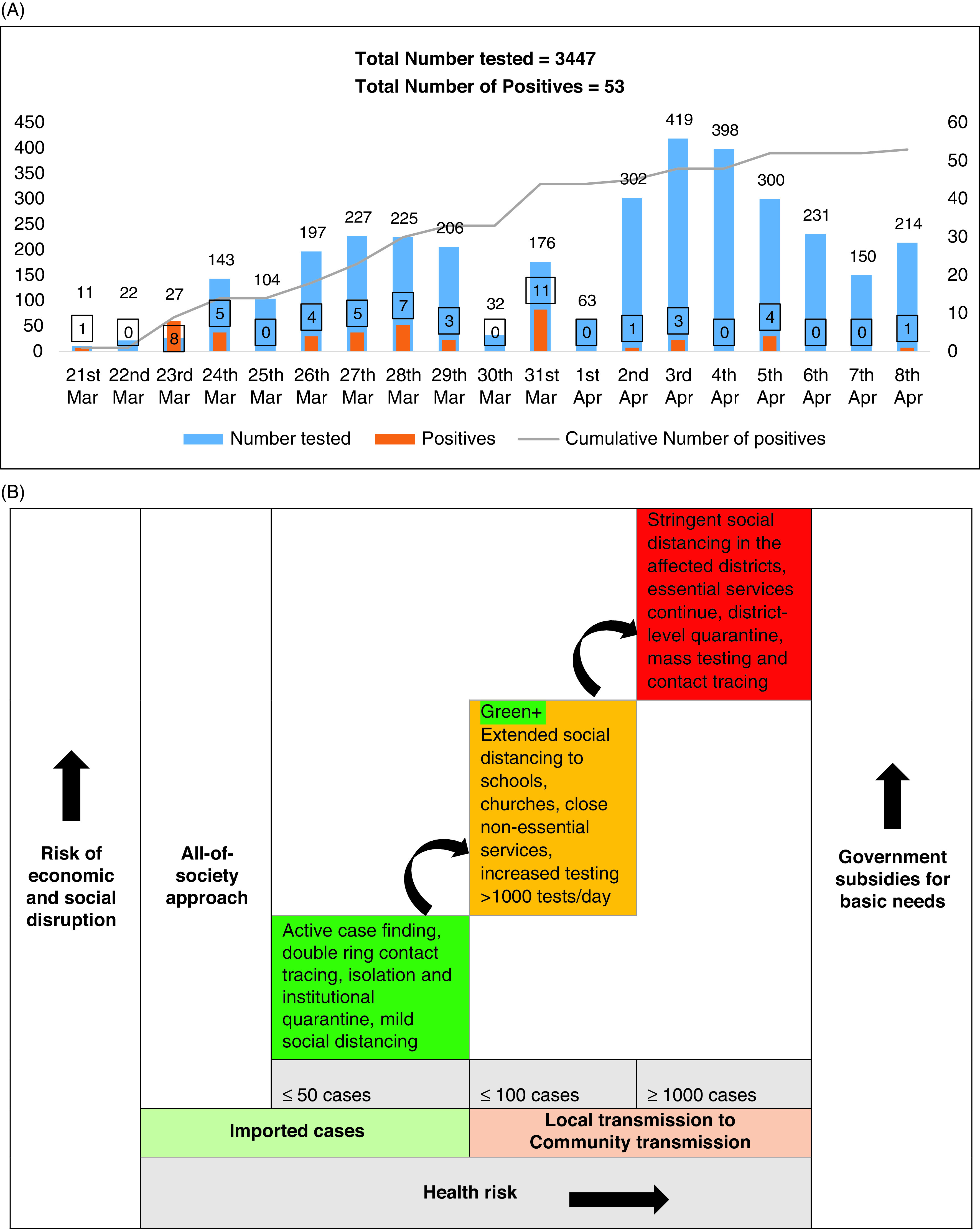
Number of COVID-19 Cases and Decision-making Matrix. (A) Number of COVID-19 tests and cases over time. (B) Rational decision-making matrix in the face of the COVID-19 pandemic.

**TABLE 1:** Number of Tests and Cases Reported to Africa CDC by June 20, 2020

	Uganda	Rwanda	Tanzania*	Kenya	Ethiopia
Number of tests	165 208	105 889	2680	133 541	207 023
Number of cases	755	661	509	4374	4070
Deaths (CFR)	0 (0)	2 (0.03)	21 (4.13)	119 (2.72)	72 (1.77)
Active cases	263	310	331	2824	3043
Incidence per 100 000 population	1.65	5.10	0.85	8.13	3.54
Health risk/ transmission classification	*Sporadic cases*	*Sporadic cases*	*Community transmission*	*Community transmission*	*Community transmission*

* Tanzania data was last updated in April

COVID-19 presents different outbreak-response dynamics in the region since it is likely to start in urban areas compared with previous viral outbreaks. The remoteness of the Ebola outbreak in Congo provides a natural quarantine for the disease, which limits spillover to towns. In contrast, COVID-19 was imported into cities, and many World Health Organization (WHO) African countries responded with border closure to prevent multiple introductions of SARS-CoV-2. By April 8, 44 of 47 countries had restricted entry into their territories; 23 of 47 countries had instituted stringent social distancing, including a curfew in 8 countries.^4^ Although stringent social distancing has been shown to halt the spread of SARS-CoV-2 in China,^5^ blind implementation of such measures in a region where 413 million people live in poverty may be counterproductive. In Uganda, the approach has been a phased implementation of restrictions. However, having a decision-making matrix enables response teams to titrate interventions against the mode of transmission, the number of active cases, and the socioeconomic situation. The matrix recommends that when the measures become stringent, authorities should provide subsidies, especially food, and allow essential activities, for example, agriculture (the main economic activity in the region), importation and exportation, and manufacturing, to operate under standard guidelines. A description of this matrix is found in Figure 1B.

In summary, the lessons learned from previous EVD outbreaks are helping countries prepare and respond to COVID-19, and the lessons from Uganda’s initial response may help other countries in the region.
